# Avian influenza infection dynamics under variable climatic conditions, viral prevalence is rainfall driven in waterfowl from temperate, south-east Australia

**DOI:** 10.1186/s13567-016-0308-2

**Published:** 2016-02-06

**Authors:** Marta Ferenczi, Christa Beckmann, Simone Warner, Richard Loyn, Kim O’Riley, Xinlong Wang, Marcel Klaassen

**Affiliations:** Centre for Integrative Ecology, School of Life and Environmental Sciences, Deakin University, Geelong, VIC 3220 Australia; Department of Economic Development, Jobs, Transport and Resources, Biosciences Research, AgriBio, Centre for AgriBiosciences, 5 Ring Road, Bundoora, VIC 3083 Australia; Department of Sustainability and Environment, Arthur Rylah Institute for Environmental Research, Heidelberg, VIC Australia; Eco Insights, 4 Roderick Close, Viewbank, VIC 3084 Australia

## Abstract

Understanding Avian Influenza Virus (AIV) infection dynamics in wildlife is crucial because of possible virus spill over to livestock and humans. Studies from the northern hemisphere have suggested several ecological and environmental drivers of AIV prevalence in wild birds. To determine if the same drivers apply in the southern hemisphere, where more irregular environmental conditions prevail, we investigated AIV prevalence in ducks in relation to biotic and abiotic factors in south-eastern Australia. We sampled duck faeces for AIV and tested for an effect of bird numbers, rainfall anomaly, temperature anomaly and long-term ENSO (El-Niño Southern Oscillation) patterns on AIV prevalence. We demonstrate a positive long term effect of ENSO-related rainfall on AIV prevalence. We also found a more immediate response to rainfall where AIV prevalence was positively related to rainfall in the preceding 3–7 months. Additionally, for one duck species we found a positive relationship between their numbers and AIV prevalence, while prevalence was negatively or not affected by duck numbers in the remaining four species studied. In Australia largely non-seasonal rainfall patterns determine breeding opportunities and thereby influence bird numbers. Based on our findings we suggest that rainfall influences age structures within populations, producing an influx of immunologically naïve juveniles within the population, which may subsequently affect AIV infection dynamics. Our study suggests that drivers of AIV dynamics in the northern hemisphere do not have the same influence at our south-east Australian field site in the southern hemisphere due to more erratic climatological conditions.

**Electronic supplementary material:**

The online version of this article (doi:10.1186/s13567-016-0308-2) contains supplementary material, which is available to authorized users.

## Introduction

Knowledge of infection dynamics is central to our understanding of zoonotic diseases, their impact on wildlife populations and the potential of these diseases to spill over into domestic animal or human populations [1]. Avian influenza virus (AIV) in its low pathogenic form, causing only mild or non-detectable clinical signs, occurs naturally in wild bird populations [2]. Recently there has been increasing interest in AIV infection dynamics, largely in response to highly pathogenic AIV outbreaks in domestic poultry and the possibility of virus transmission to humans [3].

In the northern hemisphere, AIV prevalence shows marked seasonal fluctuations in wild bird communities, with a yearly peak in late summer/early autumn, followed by low prevalence in winter [4, 5]. However the degree of seasonality varies geographically, with seasonal amplitude or intensity, tending to be lower and longer-lasting at low latitudes. In a continent-wide comparison of North American AIV data from waterfowl, Lisovski et al. (unpublished data) found a relationship between the shape of the annual infection dynamics and the degree of seasonality. Overall seasonal intensity and duration were positively correlated with geographically corresponding amplitudes and durations of the infection peak. In contrast to the northern areas, in southern North America, Lisovski et al. found much less pronounced seasonal variation in AIV infection dynamics than in comparable northern sites.

Extreme climate anomalies are observed in both hemispheres [6]. These anomalies are primarily related to the El Niño Southern Oscillation (ENSO) [6], which reflects fluctuating ocean temperatures in the east equatorial Pacific [7, 8]. Although ENSO has a nearly global effect on climate, it particularly affects precipitation patterns in the southern hemisphere with extreme rainfalls occurring in the central and eastern Pacific, Peru, Ecuador and Southern Brazil and droughts in Australia, Indonesia, India, West Africa and Northeast Brazil [6]. In Australia ENSO drives the erratic and less seasonal weather patterns that are characteristic over large parts of the continent, particularly the south-east [9, 10]. As a consequence, AIV dynamics in the southern hemisphere may differ from the widely observed seasonal dynamics in the northern hemisphere.

While tens of thousands of individual wild birds have been sampled for AIV in the northern hemisphere [11, 12], a comparatively smaller number have been sampled in Australia [13, 14] and there remains a lack of information on AIV prevalence and temporal variation in wild birds from the southern hemisphere. Based on northern hemisphere studies, the main reservoirs of AIV belong to the family *Anatidae* (swans, geese and ducks) [11, 15, 16]. The highest infection rates occur in the subfamily *Anatinae* (dabbling ducks), with nearly all AIV subtypes being found in wild dabbling ducks [4, 11]. This may be due to their “surface-feeding” behaviour, which makes dabbling ducks particularly prone to infection via the fecal-oral route [4, 17]. Furthermore, some dabbling ducks often feed on land, including farm pastures, where they may mix with domestic birds [18, 19]. As Australian dabbling duck species share many similarities in behavior and ecology with their northern hemisphere counterparts [17, 18, 20], we consider it highly likely that dabbling ducks and other Australian ducks with related ecologies, are potentially important reservoirs of AIV in Australia.

In the northern hemisphere the following three mechanisms have been suggested in maintaining the seasonality of AIV dynamics among waterbird communities: (1) the annual increase in abundance of immunologically naïve young birds results in a higher number of individuals susceptible to infection in the waterbird community [4, 16], (2) the seasonal congregation of migratory birds at staging and wintering sites increases contact rates and thereby infection rates [4, 21], and (3) migration influences an individual’s susceptibility to infection since long distance movements are energy demanding and may potentially impair immuno-competence [22]. These three hypothesized, key drivers of AIV dynamics are all linked to the annual breeding cycle of waterfowl [4].

Water availability is an important factor in the ecology of waterfowl in Australia [18, 23, 24]. Across much of the Australian continent, climatic conditions are extreme and non-seasonal [25]. Although regular rains fall seasonally in the tropics (summer) and the temperate south-east and south-west (winter-spring) regions, water availability varies largely non-seasonally across the rest of the continent. Inland areas, in particular, may lack water for longer periods. Inland Australia contains extensive flood-plains and wetland systems that may be filled by water-flows from distant rain events [26]. In south-eastern Australia inter-annual variation in rainfall is very high, with marked effects on breeding waterfowl [9]. Wet and dry periods can persist for several years [25], occasionally creating extreme climate events, such as the “Big Dry” phenomenon in south-eastern Australia between 1997 and 2009 [27]. These inter-annual and multi-year periodic climate changes are ENSO linked [9, 28]. Periods of drought across east, especially south-east Australia usually correlate with the El Niño phase of the ENSO, when the Pacific Ocean is warm and atmospheric pressure is higher than average across Australia [10]. The extreme rainfall events occur during the La Niña phase of ENSO, when the ocean is cooler and atmospheric pressure is below average [10].

These ENSO driven irregular climatic conditions strongly influence the movement and breeding biology of many Australian waterfowl at local, regional and continental scales [20, 29]. After high precipitation, bird numbers increase at flooded areas where food resources become available, creating appropriate conditions for breeding [30, 31]. During these wet periods bird numbers decrease at permanent wetlands, and the birds only return when the temporary wetlands begin to dry [9]. Klaassen et al. [32] suggested that the non-seasonal and often multi-year alternations of wet and dry periods that influence the ecology of waterfowl might therefore also affect the temporal patterns of AIV prevalence on the Australian continent. Adopting the same three hypotheses mentioned above, but now applying them to the typical climatic conditions of the Australian continent, Klaassen et al. hypothesized that intense rainfall leads to breeding events and increased numbers of immunologically naïve juvenile birds. After breeding, when the temporary wetlands dry, increasing densities of (immunologically naïve) waterbirds returning to permanent water bodies might be driving AIV prevalence in wild waterfowl in Australia. Another study in Australia showed a relationship between regional variation in rainfall and evolutionary dynamics of AIV that is possibly linked to waterbird movements and behavior [33].

To test Klaassen et al.’s hypothesis for south-east Australia, we investigated AIV prevalence in faecal samples from dabbling ducks in relation to biotic (bird numbers) and abiotic (weather) drivers at a major permanent wetland, the Melbourne Water Western Treatment Plant (WTP), 40 km from Melbourne, Australia, between 2006 and 2012.

## Materials and methods

### Faecal sampling and analysis

**Figure 1:**
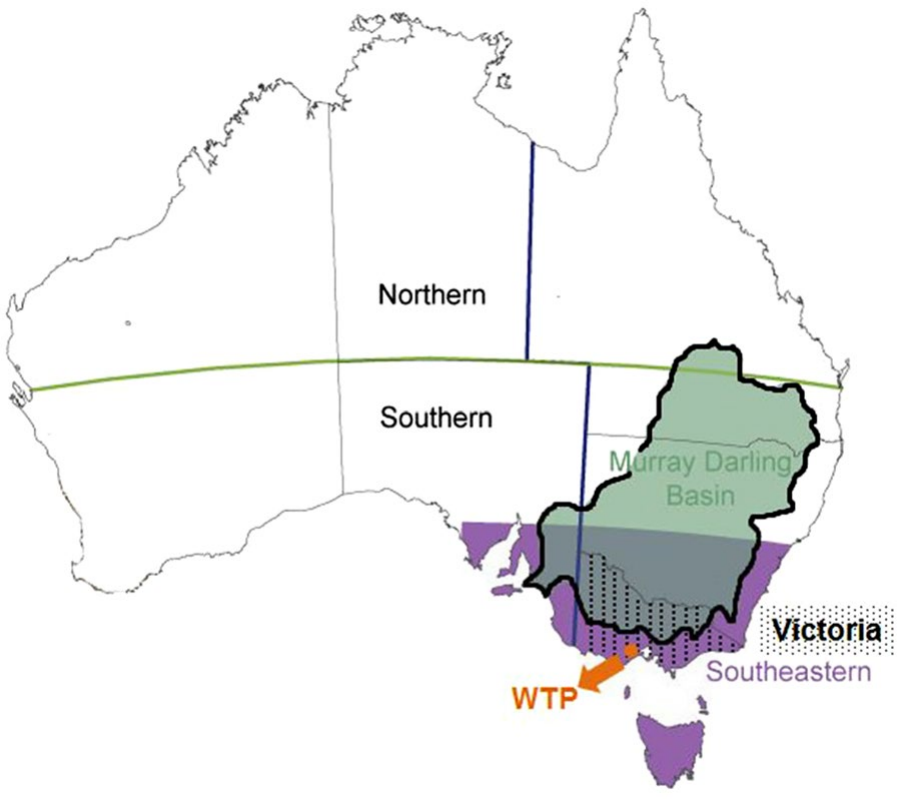
**Geographic regions.** Monthly rainfall and temperature anomalies were calculated for the following geographic regions: Western Treatment Plant, Victoria, South-eastern Australia and Murray–Darling Basin. Adapted from Australian Bureau of Meteorology.

Faecal samples for AIV analyses were collected at the WTP waste-stabilization ponds, operated by Melbourne Water in Victoria, Australia (37°59′11.62′′S, 144°39′38.66′′E). The WTP covers 10851 ha and consists of a 1820 ha array of sewerage ponds (Figure 1). This Ramsar-listed pond system is one of the most significant sites for waterbirds in Victoria, providing habitat for numerous waterfowl species that often occur in flocks of up to tens of thousands of individuals [19, 34, 35], with totals sometimes exceeding 100 000 [19, 36].

We sampled faeces of roosting waterbirds at WTP’s 115 East Lagoon, between 2006 and 2012. This site was chosen as it offers several of the few land-based roosting sites for waterbirds in the area, is closed to the public and harbours large numbers of waterbirds at all times of the year. Samples were collected at irregular intervals, with two to nine ($\bar{X} = 4.8$) collection events per year. Each collection event covered 1–3 days. Prior to faecal sample collection (i.e. before flushing the birds), waterfowl species at the roost locations were identified, with species being recorded as present/absent. As waterfowl are commonly found roosting in mixed species flocks (M. Ferenczi and M. Klaassen, personal observations), it was not possible to positively link species to individual faecal samples. Three fresh faecal samples were collected into each vial containing 4 mL viral transport media, with 13 to 365 ($\bar{X} = 96.9$) pooled samples (hereafter called “samples”) being collected per collection event. The viral transport media used was consistent with standards outlined in Johnson [37]. Samples were immediately chilled and transported to the laboratory of the Department of Economic Development in Melbourne, within 1 h drive from the study site. Samples were then stored at 4 °C until analysed for AIV presence, within 1–7 days after collection. AIV presence was tested using an influenza A PCR targeting the highly conserved matrix gene. Samples were processed for RNA extraction using 5XMagMAX-96 Viral Isolation Kit (Ambion Cat. No AMB1836-5) on a Thermo KingFisher-96 Robot. A volume of 35 μL was then run in a Superscript III Platinum One-Step qRT-PCR Kit with ROX (Life Technologies 11745-100) using a Px2 Thermal Cycler PCR machine. A volume of 5 μL (diluted 1/100) was then transferred to 15 μL Fast SYBR Green mastermix (Life Technologies 4385612) solution and the PCR performed in an ABI 7500 Real Time PCR machine. AIV prevalence with 95% CIs was calculated for each month within which a collection event took place, resulting in 34 prevalence estimates across the 7 year period.

### Bird counts

During the 2006–2012 study period regular waterbird counts were conducted as part of a longer-term study for Melbourne Water, with the total numbers of each waterbird species counted on each treatment pond [19]. These counts were carried out over a period of 1–6 days typically during the months of January, February or March, May, July, September and November. Additional counts were also conducted in June 2006 and in October 2010.

For our comparison of bird counts with AIV prevalence, we combined count data from all ponds to give the total of all birds counted over the entire WTP area. A total of 94 waterbird species were observed. For our analysis, we excluded species observed fewer than ten times during the 7 year study period. For the remaining 66 species we conducted a principal component analysis (PCA) in R [38] in an attempt to reduce the number of variables (i.e. species) into a smaller number of principal components that could account for most of the variance in the observed species numbers. We also conducted a PCA across the 13 guilds (coots, dabbling ducks, diving ducks, filter-feeding ducks, fish-eaters, grazing ducks, grebes, gulls, waterhens, large wading birds, swans, terns and waders) to which these 66 species belong. Neither the species nor guild PCA resulted in a limited number of principal components explaining a large proportion of the variation in bird numbers. For our comparison of bird counts with AIV prevalence we thus changed strategy and focused on only those species that were both abundant and frequently observed at the faecal sampling sites.

At the faecal collection sites a total of 11 species were recorded across all collection events. Ducks, and notably dabbling ducks (*Anatinae*), are thought to be the main reservoir for AIV in the northern hemisphere (see “Introduction” section), therefore we focused on duck species that were observed at the faecal collection sites: Australian Shelduck (*Tadorna tadornoides*) (3.5% of all birds recorded), Pacific Black Duck (*Anas superciliosa*) (32.6% of all birds recorded), Pink-eared Duck (*Malacorhynchus membranaceus*) (5.8% of all birds recorded) and Teal *spp* (33.7% of all birds recorded). As teal were observed as mixed flocks of Grey (*Anas gracilis*) and Chestnut Teal (*Anas castanea*), both species were included in the analysis. Of these five species, two are not dabbling ducks (Australian Shelduck and Pink-eared Duck); however, these species show many similarities to dabbling ducks in their ecology, and oropharyngeal/cloacal samples from these two species have also regularly tested positive for AIV in other projects in Australia [14]. These five selected species contributed 70.58% of the duck population at WTP at any one point in time. The Australasian Shoveler (*Anas rhynchotis*) was also frequently observed in the area, but was excluded from analysis as it was rarely observed roosting at the locations where faecal samples were collected (W. Steele personal communications), thus samples collected are unlikely to be from Australian Shovelers. Of the bird species considered, Grey Teal and Pink-eared Duck breed mainly on ephemeral wetlands in inland Australia whereas the other three have substantial local breeding populations in south-eastern Australia [9, 18, 39]. These differences produce contrasting patterns of variation in numbers of birds at permanent wetlands in south-eastern Australia [19, 40]. To allow matching of bird count and AIV data, we used linear interpolation of the bird count data for the five species to obtain monthly bird counts.

### Weather data

Monthly rainfall and temperature (anomaly) data were obtained from the Australian Bureau of Meteorology for four geographic regions of progressively increasing size: WTP (105 km^2^), Victoria (VIC) (227 600 km^2^), South-eastern Australia (SE) (723 333 km^2^) and Murray–Darling Basin (MDB) (1 056 450 km^2^) (Figure 1). For all regions except WTP, anomalies were provided online and were calculated as departures from the 1961–1990 average reference values [41]. For WTP we calculated anomalies from downloaded rainfall and temperature data using departures from the reference values in VIC (1961–1990) as rainfall and temperature data were not available for all years at WTP between 1961 and 1990.

The first 3 years of our study occurred during a period of drought, and the last 4 years during a wet period. To investigate the effect of these long-term, ENSO driven weather effects, we included an additional “ENSO drought/wet” factor in our models, which was either “dry” (2006–2009) or “wet” (2010–2012).

### Statistical analysis

#### Effects of biotic drivers (bird numbers) on AIV prevalence

To examine the effects of bird numbers (i.e. bird count data) on AIV prevalence, we used generalized linear models (GLM) weighted for total sample size (i.e. number of AIV samples collected at each collection event). A total of five GLMs were run, one for each species, with monthly AIV prevalence as the binomial response variable and bird numbers as the explanatory variable.

#### Effects of abiotic drivers (weather) on AIV prevalence

The effects of weather are not always immediately expressed in ecological processes, thus there may be a cumulative effect and a “time-lag” between changes in the weather and AIV prevalence [42, 43]. To investigate these cumulative and time-lag effects of rainfall and temperature on monthly AIV prevalence we calculated the average rainfall and temperature anomalies over the same month in which the prevalence estimate took place, and the preceding 1–12 months. Thus each monthly AIV prevalence estimate was compared with the average rainfall and temperature data from the same month and the preceding month, the same month and the preceding 2 months, same month and preceding 3 months, etc. up to 12 months. These twelve “time-lag classes” for rainfall and temperature anomalies were calculated for all four geographic regions. We tested for effects of rainfall anomalies, temperature anomalies, and “ENSO drought/wet” factor on AIV prevalence using GLMs. A total of 48 GLMs were run for all possible combinations of regions (4) and time-lag class (12) weighted for total sample size (i.e. number of AIV samples collected at each collection event). Monthly AIV prevalence as the binomial response variable was analyzed in relation to rainfall anomaly, temperature anomaly and “ENSO drought/wet” factor as explanatory variables. Within each region the best fitting model(s) among the 12 time-lag classes were selected based on their Akaike information criterion (i.e. lowest AIC value as best fit and ΔAIC < 2) [44]. Combining the above analyses in a single GLM would result in an over-parameterization of the models and result in spurious outcomes.

#### Interaction of biotic and abiotic drivers affecting AIV prevalence

To understand how bird numbers and weather data might potentially interact in ultimately explaining AIV prevalence, we analyzed the relationship between bird counts and (time-lagged) weather data in a similar fashion as outlined above using linear models (LM). A total of 240 LMs were run for all possible combinations of geographic regions (4), species (5), and time-lag class (12), in which monthly bird counts as the response variable were analyzed in relation to rainfall anomaly, temperature anomaly and “ENSO drought/wet” factor as explanatory variables. Within each region and species, the best fitting model(s) were selected based on their AIC value [44], and the significance level of explanatory variables. Selected models had to have a ΔAIC < 2 and at least one of the explanatory variables had to be significant (i.e. *p* < 0.05).

We ran the above three clusters of models rather than running full-factorial models, combining all possible combinations of abiotic and biotic factors to explain viral prevalence, since the latter resulted in over-parameterization and underidentified models [45]. We Z-transformed all variables prior to statistical analyses to allow appropriate comparison of the effect sizes (in terms of odds ratios) of the explanatory variables. After GLM and LM analyses, effect sizes of the parameter estimates were calculated as odds ratios (OR) of the Z-transformed explanatory variables. An OR > 1 indicates a positive, whereas an OR < 1 indicates a negative effect of the explanatory variable. All analyses were conducted using R [38].

## Results

**Figure 2:**
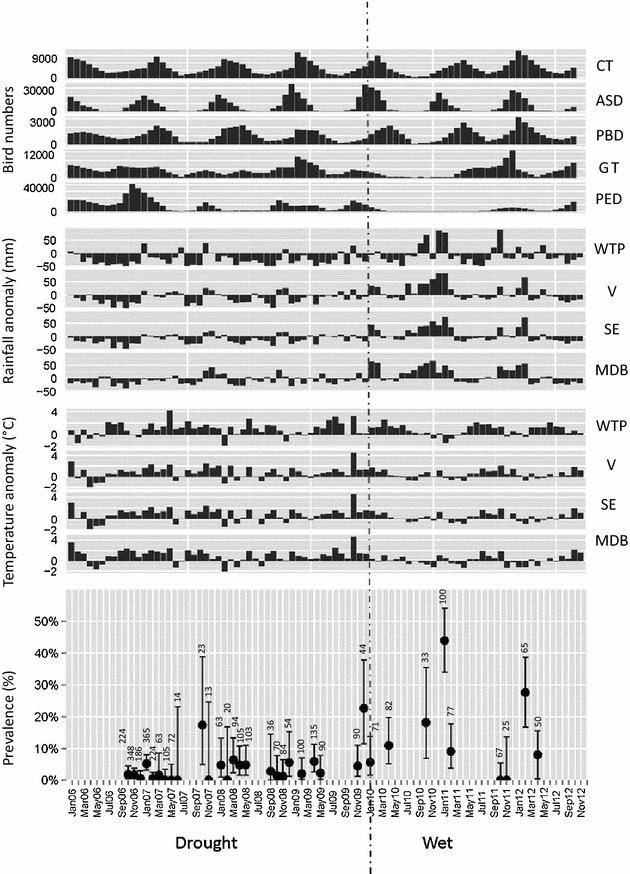
**Bird numbers of five waterfowl species, rainfall anomaly and temperature anomaly for four geographic regions, and AIV prevalence with 95% confidence intervals between 2006 and 2012.** The data is subdivided into a drought (2006–2009) and wet (2010–2012) period. The bird species for which data are presented are Chestnut Teal (CT), Australian Shelduck (ASD), Pacific Black Duck (PBD), Grey Teal (GT) and Pink-eared Duck (PED). The four geographic regions are Western Treatment Plant (WTP), Victoria (VIC), South-eastern Australia (SE) and Murray–Darling Basin (MDB).

A total of 3295 pooled faecal samples were collected, of which 179 (5.43%) tested positive for AIV. Over the 7 year period AIV prevalence varied greatly, without any apparent seasonal pattern (explicit testing of seasonality was not prudent given the many time gaps in the data; Figure 2). Bird numbers also fluctuated greatly, with some species showing clear seasonal variation in numbers (termed “seasonal” species such as Chestnut Teal, Australian Shelduck and Pacific Black Duck) and others less so (termed “non-seasonal” species such as Grey Teal and Pink-eared Duck) (Figure 2). The “seasonal” species have substantial breeding populations in temperate south-eastern Australia in permanent wetlands, whereas the “non-seasonal” species breed mainly inland where wetland availability is more erratic [18]. Rainfall and temperature data also showed high variability between years. In particular, rainfall increased in amount and frequency at the beginning of 2010 when the drought from the previous 10 years broke (Figure 2).

### Effects of biotic drivers (bird numbers) on AIV prevalence

The relationship between AIV prevalence and bird numbers varied among the five bird species (Table 1). AIV prevalence was significantly positively related to Australian Shelduck numbers, significantly negatively related to Grey Teal and Pink-eared Duck, and not correlated with Chestnut Teal and Pacific Black Duck numbers (Table 1).

**Table 1:** Odds ratios (OR) and AICs for the best fitting generalized linear models of the effects of bird number of five waterfowl species on AIV prevalence

Species	Seasonality (breeding area)	Species number OR	AIC
Australian Shelduck	Seasonal (south-eastern Australia)	1.91***	322.11
Chestnut teal	Seasonal (south-eastern Australia)	1.09	383.60
Grey teal	Non-seasonal (inland)	0.40***	272.49
Pacific black duck	seasonal (south-eastern Australia)	0.94	384.07
Pink-eared duck	Non-seasonal (inland)	0.56***	316.13

An OR > 1 indicates a positive, whereas an OR < 1 indicates a negative effect of the explanatory variable (i.e. OR > 1 means that AIV prevalence was greater when bird numbers were higher and OR < 1 means that AIV prevalence was greater when bird numbers were lower). Stars indicate significance level: ***p < 0.001.

### Effects of abiotic drivers (weather) on AIV prevalence

Out of the 48 models that examined AIV prevalence in relation to rainfall anomalies, temperature anomalies and “ENSO drought/wet” factor for the various regions and time-lag classes, seven models were found to have a ΔAIC < 2. In these seven best models, rainfall anomalies had a significant and substantial positive effect on AIV prevalence (Table 2). This significant result also held true for most of the remaining models [OR 1.43–2.48; (Additional file 1)]. Five of the seven best models in WTP, VIC and SE also showed a significant positive effect of “ENSO drought/wet” factor on prevalence, wet years being associated with a higher AIV prevalence (Figure 2, Table 2). This also held true for 18 of the remaining models (Additional file 1).

**Table 2:** Odds ratios (OR) and AICs for the best fitting generalized linear models describing the effects of rainfall anomaly, temperature anomaly and “ENSO drought/wet” factor on AIV prevalence

Time-lag class	Region	Rainfall anomaly OR	Temperature anomaly OR	“ENSO drought/wet” OR	AIC
3	WTP	1.55***	0.88	1.49***	228.27
5	VIC	1.67***	0.97	1.35**	231.33
4	SE	1.59***	0.88	1.37**	234.69
5	SE	1.64***	0.95	1.36**	234.16
6	SE	1.74***	1.07	1.32*	233.32
7	SE	1.80***	1.04	1.23	235.01
6	MDB	2.48***	1.34*	1.08	213.16

An OR > 1 indicates a positive, whereas an OR < 1 indicates a negative effect of the explanatory variable (e.g. OR > 1 means that AIV prevalence was greater when rainfall anomaly was higher and OR < 1 means that AIV prevalence was greater when rainfall anomaly was lower). Regions: WTP (Western Treatment Plant), Victoria (VIC), South-eastern Australia (SE) and Murray–Darling Basin (MDB). Stars indicate significance levels: ***p < 0.001; **p < 0.01; *p < 0.05.

Temperature anomaly had a significant positive effect on AIV prevalence in only one of the seven best models (Table 2). In the remaining models, the effect of temperature anomalies on AIV prevalence had a mix of significant positive and negative effects (Additional file 1).

Investigating the time-lag classes of the models, we found that environmental conditions averaged over the preceding 3 months to preceding 7 months provided the best model fits (Table 2).

### Interaction of biotic and abiotic drivers affecting AIV prevalence

The positive effect of these short and long-term rainfall patterns on AIV prevalence described above is likely mediated through a complex interaction with bird numbers. We found that numbers of “seasonal” species that breed in south-eastern Australia were either positively (Australian Shelduck), or not (Chestnut Teal and Pacific Black Duck) related to AIV prevalence (Table 1). Numbers of these duck species did not show any (consistent) patterns in numbers in relation to climatic variables (Table 3, Additional file 2). “Seasonal” bird numbers were not affected by “ENSO drought/wet” factor and mostly unaffected by rainfall anomalies (with positive and negative effects in only a few cases). Temperature anomalies had mainly mixed positive and negative effects on “seasonal” bird numbers (Table 3, Additional file 2). These results were apparent across all time-lag classes (i.e. all 240 models tested; Additional file 2), including the selected best models (Table 3).

**Table 3:** Odds ratios (OR) and AICs for the best fitting linear models describing the effects of rainfall anomaly, temperature anomaly and “ENSO drought/wet” factor on bird number of five waterfowl species

Time-lag class	Region	Species	Rainfall anomaly OR	Temperature anomaly OR	“ENSO drought/wet” OR	AIC
3	WTP	Chestnut Teal	1.23	0.65*	0.83	95.25
4	WTP	Chestnut Teal	1.22	0.63**	0.82	94.06
6	VIC	Chestnut Teal	1.03	1.45*	1.02	100.44
7	VIC	Chestnut Teal	0.97	1.48*	1.07	99.94
8	VIC	Chestnut Teal	0.85	1.44*	1.17	100.34
–	SE	Chestnut Teal	–	–	–	–
12	MDB	Chestnut Teal	0.49*	0.60*	1.40	99.68
1	WTP	Australian Shelduck	1.09	0.64*	1.11	95.18
2	WTP	Australian Shelduck	1.27	0.70*	1.04	95.20
3	WTP	Australian Shelduck	1.25	0.69*	1.05	94.75
–	VIC	Australian Shelduck	–	–	–	–
–	SE	Australian Shelduck	–	–	–	–
4	MDB	Australian shelduck	1.94*	1.65*	0.99	96.13
5	MDB	Australian Shelduck	1.81*	1.74*	1.05	95.18
4	WTP	Pacific black Duck	1.12	0.63*	0.85	96.20
5	WTP	Pacific black Duck	1.26	0.68*	0.81	96.24
6	WTP	Pacific black Duck	1.27	0.67*	0.81	95.87
6	VIC	Pacific black Duck	1.01	1.48*	1.02	99.89
7	VIC	Pacific black Duck	0.90	1.53*	1.11	98.52
8	VIC	Pacific black Duck	0.77	1.49*	1.24	98.63
–	SE	Pacific black Duck	–	–	–	–
1	MDB	Pacific black Duck	0.77	0.58**	1.00	95.08
2	MDB	Pacific black Duck	0.83	0.56**	0.94	95.08
12	WTP	Grey Teal	0.81	0.52***	0.73	76.85
11	VIC	Grey Teal	0.63	0.61**	0.97	84.59
12	VIC	Grey Teal	0.74	0.58***	0.85	83.29
11	SE	Grey Teal	0.57*	0.60***	0.99	84.38
12	SE	Grey Teal	0.65	0.56***	0.86	82.86
11	MDB	Grey Teal	0.34***	0.51***	1.23	83.39
12	MDB	Grey teal	0.37***	0.48***	1.10	84.71
11	WTP	Pink-eared Duck	0.60*	0.60***	0.96	80.33
9	VIC	Pink-eared Duck	0.42***	0.66***	1.32	72.29
9	SE	Pink-eared Duck	0.38***	0.70**	1.40	74.99
9	MDB	Pink-eared Duck	0.32***	0.69*	1.44	79.13

An OR > 1 indicates a positive, whereas an OR < 1 indicates a negative effect of the explanatory variable (e.g. OR > 1 means that bird numbers were higher when rainfall anomaly was higher and OR < 1 means that bird numbers were higher when rainfall anomaly was lower). Regions: WTP (Western Treatment Plant), Victoria (VIC), South-eastern Australia (SE) and Murray–Darling Basin (MDB). Stars indicate significance levels: ***p < 0.001; **p < 0.01; *p < 0.05. Empty cells indicate that the models were not significant for that region and species, thus best models could not been selected.

The “non-seasonal” species (i.e. Grey Teal and Pink-eared Duck) that tend to move long distances and breed inland, were negatively related to AIV prevalence (Table 1). Duck numbers were negatively related to rainfall and temperature (Table 3, Additional file 2), with numbers at the WTP dropping when the wet period started (2010), suggesting they indeed left the area to breed inland (see Figure 2). For the “seasonal” species, the best models were apparent across almost all time-lag classes, yet the best models for “non-seasonal” species were restricted to 9, 11, and 12 month time-lag classes (i.e. conditions averaged over the preceding 9, 11 and 12 months; see Table 3).

**Figure 3:**
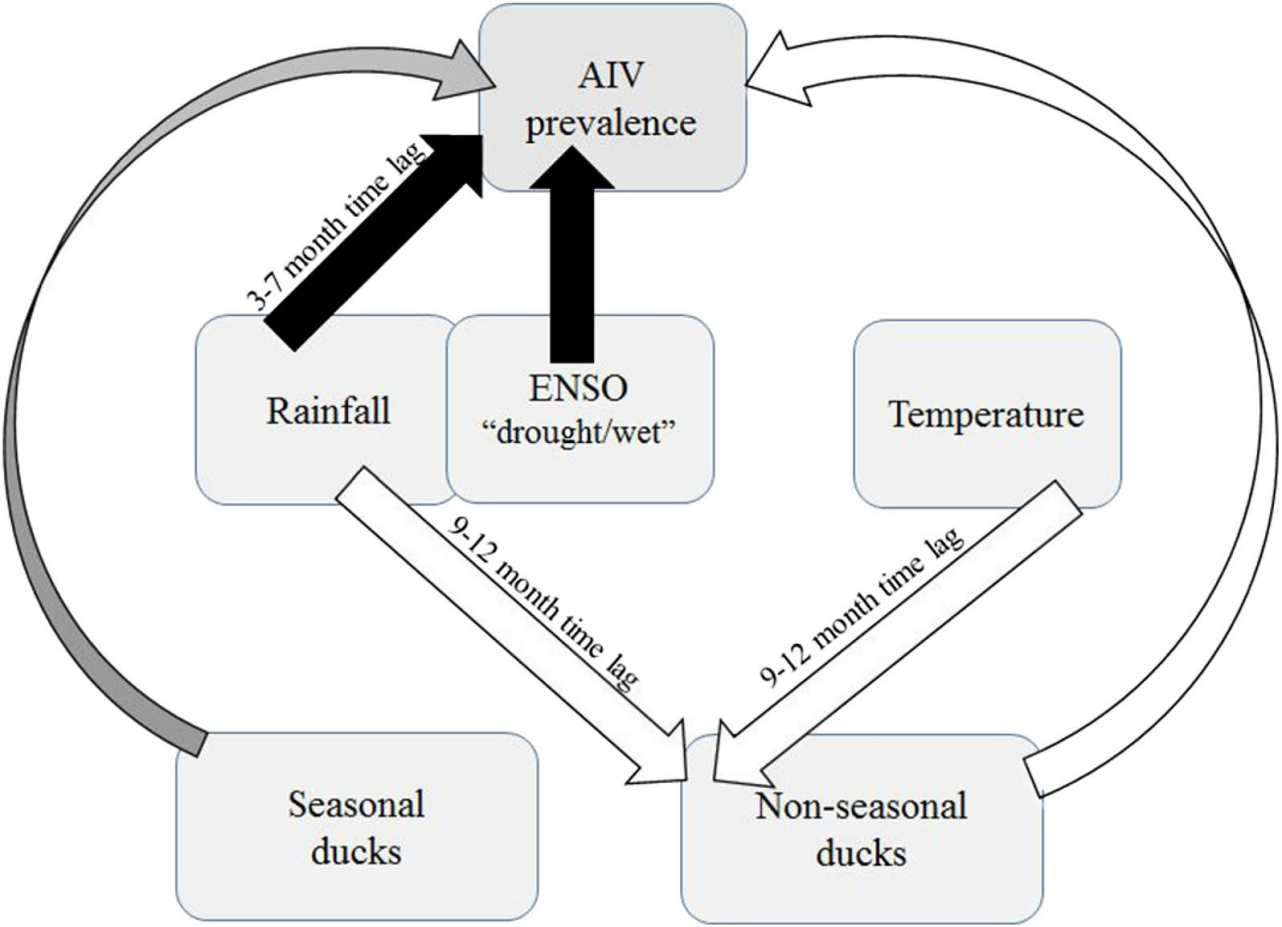
**Diagram summarizing the main relationships between climate factors, bird numbers and AIV prevalence.** Arrows show the direction of the effects, including potential time-lags. The colour of the arrows indicates the direction of the correlations and whether the relationship is direct or indirect (white—direct negative effect; grey—mixed direct effects (positive effect of one species and no effects for the two other species); black—positive indirect effect). The close link between rainfall and ENSO drought/wet is reflected by their partial overlap in the diagram.

An integrated summary of the results is presented in a schematic diagram in Figure 3, including an interpretation of the correlations found into possible direct and indirect effects.

## Discussion

We found AIV prevalence among wild dabbling ducks to be related to rainfall patterns. This was apparent at two temporal scales: as a positive effect of long term rainfall patterns (“ENSO drought/wet” factor) that was linked to the wet period 2010–2012, and as a more immediate positive response (albeit with time-lag effects of three to 7 months; i.e. best results were obtained when considering rainfall in the preceding 3–7 months). ENSO and rainfall in SE Australia are closely linked [9] and the apparent “ENSO drought/wet” effect reinforces the importance of the indirect effect of rainfall on AIV dynamics rather, than an effect of ENSO per se. The occasional lack of an ENSO drought/wet effect on AIV prevalence at larger geographical scales (i.e. MDB and SE) is probably due to ENSO’s effects being already explained by rainfall anomalies. AIV prevalence showed a clear positive relationship with duck numbers for only one “seasonal” species (Australian Shelduck) and was not related to numbers of the remaining two “seasonal” species (Chestnut Teal and Pacific Black Duck). Numbers of both “non-seasonal” duck species (Grey Teal and Pink-eared Duck) negatively affected AIV prevalence, thus AIV prevalence at the WTP was highest when these species were away breeding (i.e. when their numbers were low at the WTP). As discussed below, rainfall patterns importantly determine breeding opportunities and are therefore linked to bird numbers [19]. Thus rainfall can influence age structures within the duck community, which may subsequently affect AIV dynamics. However, we cannot rule out that rainfall may also have a direct effect on AIV dynamics, as AIV is generally highly persistent in water [46].

In contrast to the northern hemisphere, a determinative feature of the southern hemisphere climate is the ENSO linked irregularity in both timing and location of wet and dry periods [6, 29, 39]. These erratic climate patterns may relax seasonality in breeding, where reproduction occurs during periods of higher rainfall and associated increases in food availability [18, 20, 23]. For some “seasonal” Australian ducks this means that the annual time window within which breeding can take place is much wider in wet years than what is typically found in northern hemisphere birds (5–7 vs. 3 months respectively) (e.g. Pacific Black Duck and Chestnut Teal [18]). For other “non-seasonal” species breeding may be completely opportunistic, and take place at any time of the year after intense rainfall, with multiple broods per breeding season possible (e.g. Grey Teal and Pink-eared Duck [18]). Pink-eared Ducks may begin to breed 8–28 days after intense rainfall [18]. Gonadal development in Grey Teal takes place ~60 days prior to breeding after intense rainfall [47, 48]. In our data, the relationship with rainfall for these “non-seasonal”, inland breeding ducks is evident. However, the best models indicate that their numbers decrease at the WTP in relation to rainfall averaged over the preceding 9 months to preceding 12 months after inland rain (Table 3), with the remaining models also showing significant results with rainfall averaged over the preceding 2 to preceding 12 months (Additional file 2).

We included temperature in our analysis as in the northern hemisphere AIV prevalence has been shown to increase during the colder months [49] and AIV survival has generally been shown to be negatively related to temperature [50]. However, we found no support for such an effect in our data; AIV prevalence was largely unrelated or, in one case only, positively rather than negatively related to temperature. However, we did find a negative relationship between temperature and “non-seasonal” bird numbers, suggesting that at higher temperature (with 9–11 month time-lag effects) “non-seasonal” duck species leave the coastal area and travel inland to breed. Although not as strong as the effect of rainfall (which may increase nutrient input into wetlands and also has a strong positive effect on total wetland area (for MDB during the period of our study r = 0.68) [51], relatively high temperatures may boost wetland primary productivity and thereby improve conditions for breeding [52].

Our results indicate that AIV dynamics are not simply a function of bird numbers. This is notably true for the “non-seasonal” species (Grey Teal and Pink-eared Duck), which show a negative relationship between AIV prevalence and bird numbers. The negative relationship between AIV prevalence and “non-seasonal” bird numbers overlapping in time (i.e. overlapping time-lags) with the positive relationship between rainfall and AIV prevalence suggests that the highest AIV prevalence might not be observed when numbers of “non-seasonal” species are at their highest. As we discuss below, age-structure is a key element of our hypothesis and the bird numbers per se do not reflect the ratio of adults and juveniles. This may be the result of different arrival time of juveniles and adults in the area (i.e. after breeding, inland juveniles return earlier, while adults remain inland to have second clutches). In the northern hemisphere juvenile birds have been identified as possible drivers for AIV, having higher virus isolation frequency than adults [16]. Van Dijk et al. also showed that the AIV peak in a Mallard (*Anas platyrhynchos*) population was driven by juvenile birds in the summer as they shed more viruses and also antibodies against AIV were barely detectable in their blood, in contrast to adults [14]. Although we lack data on the proportion of juveniles in the monitored populations, we suggest that an influx of juveniles that arrive from inland areas together with more locally hatched juvenile birds, is the likely a driver of AIV prevalence dynamics at our study site. This would be consistent with the time-lag of 3–7 months between rainfall and AIV prevalence. Both Pink-eared Duck and Grey Teal have a ~26–28 day incubation period and typically require ~55 days to complete total body and feather growth of ducklings [18]. Thus the first juvenile birds might be expected to arrive at the WTP 3–5 months after significant rainfall. In other waterfowl species, for example Pacific Black Duck and Australian Shelduck, breeding is also related to rainfall. In these species there is typically a 60–90 day time-lag between intense rainfall and onset of laying [23], yielding increased juvenile numbers 4–6 months after rainfall. In summary, for all duck species combined, following rainfall it takes 3–6 months before fully grown juvenile birds appear in the population. This coincides with rainfall calculated over the preceding 3–7 months being positively related to AIV prevalence in our data.

The positive relationship between resident Australian Shelduck numbers and AIV prevalence is possibly related to the increase in juveniles along with total numbers for this species. The negative relationship between numbers of inland breeding species and AIV prevalence possibly indicates that adults may remain breeding in inland areas engaging in multiple brooding, while birds present at WTP are mainly juveniles that are unlikely to breed in their first year. Young birds are known to form high proportions of the Victorian duck population in wet years (up to 80%, usually 50 or 0% in dry years) [53, 54]. Unfortunately, no data are available on juvenile percentages in the various duck populations at the WTP, necessary to confirm our hypothesis.

Analyses of AIV dynamics in other areas of the world characterized by erratic climatic conditions are, to our knowledge, not available. Several studies, however, have highlighted the importance of climate variability in driving infectious disease prevalence in humans, domestic animals, and wildlife [55–57]. Viral disease outbreak cases have also been linked to ENSO driven weather anomalies [57]. In some cases, the outbreaks were associated with drought conditions [e.g. dengue fever, 58] while in others heavy rains triggered elevated disease risk (e.g. West Nile virus) [59]. In Australia, studies focusing on viral diseases also found strong links between rainfall patterns and disease risk. In south-eastern Australia, heavy rainfall in summer and autumn increased disease risk of Murray Valley encephalitis [60]. Similarly, in south-eastern Australia Ross river viral disease was related to high summer and winter rainfalls [61]. All of the above mentioned viruses are arboviruses for which mosquitos act as a vector, their ecology being directly related to weather factors, notably precipitation [61]. Yet, besides breeding and survival of arthropod vectors, the population dynamics of potential host mammals and birds are also affected by ENSO driven weather anomalies [23, 43, 61]. Such climate driven changes in host population numbers, age structures and body condition may also play a role in the temporal patterns in disease dynamics [62, 63]. This parallels our suggestion that intense rainfall events affect the breeding ecology of waterbirds and concomitantly AIV prevalence.

Our study highlights the importance of investigating disease dynamics in various regions of the world with contrasting climatic conditions. Such a comparative approach will allow us to better identify the role of hypothesized drivers. In addition, and notably for systems regularly experiencing extreme weather events, such studies may allow for an improved evaluation of the consequences of climate change on disease dynamics [55].

## Additional files

10.1186/s13567-016-0308-2 **Odds ratios (OR) and AICs for generalized linear models of the effects of rainfall anomaly, temperature anomaly and “ENSO drought/wet” factor on AIV prevalence.** An OR > 1 indicates a positive, whereas an OR < 1 indicates a negative effect of the explanatory variable (e.g. OR > 1 means that AIV prevalence was greater when rainfall anomaly was higher and OR < 1 means that AIV prevalence was higher when rainfall anomaly was lower). Regions: WTP (Western Treatment Plant), Victoria (VIC), South-eastern Australia (SE) and Murray–Darling Basin (MDB). Stars indicate the difference between the significance levels: *** = *p* < 0.001; ** = *p* < 0.01; * = *p* < 0.05. Bold numbers indicate the best fitting models (ΔAIC < 2).
10.1186/s13567-016-0308-2 **Odds ratios (OR) and AICs for linear models describing the effects of rainfall anomaly, temperature anomaly and “ENSO drought/wet” factor on bird number for five waterfowl species.** An OR > 1 indicates a positive, whereas an OR < 1 indicates a negative effect of the explanatory variable (e.g. OR > 1 means that bird numbers were higher when rainfall anomaly was higher and OR < 1 means that bird numbers were higher when rainfall anomaly was lower). Regions: WTP (Western Treatment Plant), Victoria (VIC), South-eastern Australia (SE) and Murray–Darling Basin (MDB). Stars indicate the different significance levels of the effects: *** = *p* < 0.001; ** = *p* < 0.01; * = *p* < 0.05. Bold numbers indicate the best fitting models (ΔAIC < 2 and at least one of the explanatory variable is significant).

## Abbreviations

AIV: Avian influenza virus
ENSO: El Niño Southern Oscillation
WTP: Western Treatment Plant
PCR: polymerase chain reaction
qRT-PCR: real-time quantitative reverse transcription pcr
PCA: principal component analysis
VIC: Victoria
SE: South-eastern Australia
MDB: Murray–Darling basin
GLM: generalized linear model
LM: linear model
AIC: Akaike information criterion
OR: odds ratio
