# Supplementary material for: Avian influenza infection dynamics under variable climatic conditions, viral prevalence is rainfall driven in waterfowl from temperate, south-east Australia
Source: Vet Res. 2016 Feb 6;47:23. doi: 10.1186/s13567-016-0308-2 (PMC4744453; doi:10.1186/s13567-016-0308-2)
Supplement: Supplementary file 1 — 10.1186/s13567-016-0308-2 Odds ratios (OR) and AICs for generalized linear models of the effects of rainfall anomaly, temperature anomaly and “ENSO drought/wet” factor on AIV prevalence. An OR > 1 indicates a positive, whereas an OR < 1 indicates a negative effect of the explanatory variable (e.g. OR > 1 means that AIV prevalence was greater when rainfall anomaly was higher and OR < 1 means that AIV prevalence was higher when rainfall anomaly was lower). Regions: WTP (Western Treatment Plant), Victoria (VIC), South-eastern Australia (SE) and Murray–Darling Basin (MDB). Stars indicate the difference between the significance levels: *** = p < 0.001; ** = p < 0.01; * = p < 0.05. Bold numbers indicate the best fitting models (ΔAIC < 2). [file 13567_2016_308_MOESM1_ESM.docx]

| Time lag class | Region | Rainfall anomaly OR | Temperature anomaly OR | ENSO  OR | AIC |
| --- | --- | --- | --- | --- | --- |
| 1 | WTP | 1.15 | 0.74** | 1.92*** | 252.90 |
| 2 | WTP | 1.43*** | 0.79* | 1.63*** | 234.89 |
| 3 | WTP | 1.55*** | 0.88 | 1.49*** | **228.27** |
| 4 | WTP | 1.61*** | 0.95 | 1.45*** | 231.59 |
| 5 | WTP | 1.94*** | 1.15 | 1.27 | 230.74 |
| 6 | WTP | 1.89*** | 1.27* | 1.32* | 238.80 |
| 7 | WTP | 1.94*** | 1.21* | 1.23 | 236.90 |
| 8 | WTP | 1.84*** | 1.20* | 1.25 | 241.84 |
| 9 | WTP | 1.80 | 1.23 | 1.27 | 246.03 |
| 10 | WTP | 1.76*** | 1.28** | 1.31* | 244.87 |
| 11 | WTP | 1.59*** | 1.26** | 1.45** | 253.35 |
| 12 | WTP | 1.48*** | 1.20* | 1.59*** | 259.66 |
| 1 | VIC | 1.55*** | 0.93 | 1.52*** | 239.71 |
| 2 | VIC | 1.55*** | 0.91 | 1.49*** | 242.71 |
| 3 | VIC | 1.58*** | 0.92 | 1.43** | 238.21 |
| 4 | VIC | 1.55*** | 0.89 | 1.43** | 236.84 |
| 5 | VIC | 1.67*** | 0.97 | 1.35** | **231.33** |
| 6 | VIC | 1.66*** | 1.06 | 1.36** | 237.03 |
| 7 | VIC | 1.75*** | 1.06 | 1.26 | 237.07 |
| 8 | VIC | 1.80*** | 1.06 | 1.22 | 240.00 |
| 9 | VIC | 1.83*** | 1.04 | 1.20 | 244.34 |
| 10 | VIC | 1.75*** | 1.08 | 1.26 | 249.86 |
| 11 | VIC | 1.70*** | 1.12 | 1.31* | 250.96 |
| 12 | VIC | 1.70*** | 1.17 | 1.34* | 251.18 |
| 1 | SE | 1.64*** | 0.88 | 1.42** | 236.42 |
| 2 | SE | 1.55*** | 0.87 | 1.43** | 242.43 |
| 3 | SE | 1.60*** | 0.89 | 1.38** | 237.08 |
| 4 | SE | 1.59*** | 0.88 | 1.37** | **234.69** |
| 5 | SE | 1.64*** | 0.95 | 1.36** | **234.16** |
| 6 | SE | 1.74*** | 1.07 | 1.32* | **233.32** |
| 7 | SE | 1.80*** | 1.04 | 1.23 | **235.01** |
| 8 | SE | 1.91*** | 1.05 | 1.16 | 237.31 |
| 9 | SE | 1.84*** | 1.01 | 1.19 | 244.04 |
| 10 | SE | 1.81*** | 1.04 | 1.22 | 248.76 |
| 11 | SE | 1.85*** | 1.11 | 1.22 | 247.44 |
| 12 | SE | 1.79*** | 1.20* | 1.30* | 250.85 |
| 1 | MDB | 1.97*** | 0.79* | 1.13 | 218.37 |
| 2 | MDB | 1.94*** | 0.78* | 1.07 | 218.61 |
| 3 | MDB | 1.96*** | 0.92 | 1.13 | 222.39 |
| 4 | MDB | 2.06*** | 1.04 | 1.14 | 223.92 |
| 5 | MDB | 1.88*** | 1.01 | 1.23 | 228.17 |
| 6 | MDB | 2.48*** | 1.34* | 1.08 | **213.16** |
| 7 | MDB | 2.29*** | 1.20 | 1.09 | 220.62 |
| 8 | MDB | 2.11*** | 1.08 | 1.12 | 229.31 |
| 9 | MDB | 2.03*** | 1.06 | 1.15 | 234.09 |
| 10 | MDB | 2.22*** | 1.11 | 1.07 | 229.20 |
| 11 | MDB | 2.30*** | 1.24 | 1.09 | 231.42 |
| 12 | MDB | 2.14*** | 1.30* | 1.23 | 243.63 |
