# Supplementary material for: Avian influenza infection dynamics under variable climatic conditions, viral prevalence is rainfall driven in waterfowl from temperate, south-east Australia
Source: Vet Res. 2016 Feb 6;47:23. doi: 10.1186/s13567-016-0308-2 (PMC4744453; doi:10.1186/s13567-016-0308-2)
Supplement: Supplementary file 2 — 10.1186/s13567-016-0308-2 Odds ratios (OR) and AICs for linear models describing the effects of rainfall anomaly, temperature anomaly and “ENSO drought/wet” factor on bird number for five waterfowl species. An OR > 1 indicates a positive, whereas an OR < 1 indicates a negative effect of the explanatory variable (e.g. OR > 1 means that bird numbers were higher when rainfall anomaly was higher and OR < 1 means that bird numbers were higher when rainfall anomaly was lower). Regions: WTP (Western Treatment Plant), Victoria (VIC), South-eastern Australia (SE) and Murray–Darling Basin (MDB). Stars indicate the different significance levels of the effects: *** = p < 0.001; ** = p < 0.01; * = p < 0.05. Bold numbers indicate the best fitting models (ΔAIC < 2 and at least one of the explanatory variable is significant). [file 13567_2016_308_MOESM2_ESM.docx]

| Time lag class | Region | Species | Rainfall anomaly OR | Temperature anomaly OR | ENSO OR | AIC |
| --- | --- | --- | --- | --- | --- | --- |
| 1 | WTP | Chestnut Teal | 0.92 | 0.77 | 1.01 | 103.29 |
| 2 | WTP | Chestnut Teal | 1.09 | 0.69* | 0.91 | 99.73 |
| 3 | WTP | Chestnut Teal | 1.23 | 0.65* | 0.83 | **95.25** |
| 4 | WTP | Chestnut Teal | 1.22 | 0.63** | 0.82 | **94.06** |
| 5 | WTP | Chestnut Teal | 1.28 | 0.69* | 0.83 | 96.65 |
| 6 | WTP | Chestnut Teal | 1.25 | 0.70 | 0.84 | 98.03 |
| 7 | WTP | Chestnut Teal | 1.37 | 0.81 | 0.80 | 101.38 |
| 8 | WTP | Chestnut Teal | 1.34 | 0.94 | 0.81 | 103.99 |
| 9 | WTP | Chestnut Teal | 1.04 | 1.00 | 0.99 | 105.45 |
| 10 | WTP | Chestnut Teal | 0.86 | 1.05 | 1.15 | 105.11 |
| 11 | WTP | Chestnut Teal | 0.71 | 1.00 | 1.32 | 103.97 |
| 12 | WTP | Chestnut Teal | 0.85 | 0.90 | 1.13 | 104.72 |
| 1 | VIC | Chestnut Teal | 1.01 | 0.99 | 1.00 | 105.46 |
| 2 | VIC | Chestnut Teal | 1.16 | 1.03 | 0.92 | 105.01 |
| 3 | VIC | Chestnut Teal | 1.27 | 1.15 | 0.88 | 103.58 |
| 4 | VIC | Chestnut Teal | 1.14 | 1.27 | 0.96 | 102.99 |
| 5 | VIC | Chestnut Teal | 1.10 | 1.40 | 0.97 | 101.15 |
| 6 | VIC | Chestnut Teal | 1.03 | 1.45* | 1.02 | **100.44** |
| 7 | VIC | Chestnut Teal | 0.97 | 1.48* | 1.07 | **99.94** |
| 8 | VIC | Chestnut Teal | 0.85 | 1.44* | 1.17 | **100.34** |
| 9 | VIC | Chestnut Teal | 0.72 | 1.28 | 1.34 | 102.22 |
| 10 | VIC | Chestnut Teal | 0.65 | 1.13 | 1.45 | 102.90 |
| 11 | VIC | Chestnut Teal | 0.63 | 0.98 | 1.48 | 102.78 |
| 12 | VIC | Chestnut Teal | 0.72 | 0.90 | 1.31 | 103.73 |
| 1 | SE | Chestnut Teal | 1.06 | 0.88 | 0.95 | 104.87 |
| 2 | SE | Chestnut Teal | 1.25 | 0.91 | 0.86 | 104.05 |
| 3 | SE | Chestnut Teal | 1.39 | 1.03 | 0.82 | 103.39 |
| 4 | SE | Chestnut Teal | 1.29 | 1.14 | 0.88 | 103.82 |
| 5 | SE | Chestnut Teal | 1.25 | 1.31 | 0.92 | 102.44 |
| 6 | SE | Chestnut Teal | 1.19 | 1.37 | 0.97 | 101.70 |
| 7 | SE | Chestnut Teal | 1.09 | 1.40 | 1.02 | 101.38 |
| 8 | SE | Chestnut Teal | 0.95 | 1.37 | 1.12 | 102.02 |
| 9 | SE | Chestnut Teal | 0.81 | 1.20 | 1.25 | 103.85 |
| 10 | SE | Chestnut Teal | 0.70 | 1.04 | 1.36 | 104.05 |
| 11 | SE | Chestnut Teal | 0.65 | 0.90 | 1.42 | 103.07 |
| 12 | SE | Chestnut Teal | 0.73 | 0.82 | 1.26 | 103.26 |
| 1 | MDB | Chestnut Teal | 0.91 | 0.71 | 0.96 | 101.69 |
| 2 | MDB | Chestnut Teal | 1.06 | 0.71 | 0.87 | 101.16 |
| 3 | MDB | Chestnut Teal | 1.24 | 0.79 | 0.80 | 101.53 |
| 4 | MDB | Chestnut Teal | 1.41 | 0.95 | 0.78 | 102.70 |
| 5 | MDB | Chestnut Teal | 1.74 | 1.26 | 0.76 | 101.37 |
| 6 | MDB | Chestnut Teal | 1.72 | 1.35 | 0.80 | 100.88 |
| 7 | MDB | Chestnut Teal | 1.62 | 1.37 | 0.81 | 101.51 |
| 8 | MDB | Chestnut Teal | 1.51 | 1.35 | 0.82 | 102.62 |
| 9 | MDB | Chestnut Teal | 1.24 | 1.14 | 0.90 | 104.93 |
| 10 | MDB | Chestnut Teal | 0.84 | 0.90 | 1.11 | 105.11 |
| 11 | MDB | Chestnut Teal | 0.58 | 0.69 | 1.33 | 102.15 |
| 12 | MDB | Chestnut Teal | 0.49* | 0.60* | 1.40 | **99.68** |
| 1 | WTP | Australian Shelduck | 1.09 | 0.64* | 1.11 | **95.18** |
| 2 | WTP | Australian Shelduck | 1.27 | 0.70* | 1.04 | **95.20** |
| 3 | WTP | Australian Shelduck | 1.25 | 0.69* | 1.05 | **94.75** |
| 4 | WTP | Australian Shelduck | 1.22 | 0.75 | 1.08 | 98.10 |
| 5 | WTP | Australian Shelduck | 1.13 | 0.86 | 1.16 | 101.87 |
| 6 | WTP | Australian Shelduck | 1.09 | 0.93 | 1.20 | 102.84 |
| 7 | WTP | Australian Shelduck | 1.09 | 0.98 | 1.20 | 103.16 |
| 8 | WTP | Australian Shelduck | 0.96 | 1.06 | 1.32 | 103.18 |
| 9 | WTP | Australian Shelduck | 0.77 | 1.09 | 1.58 | 102.21 |
| 10 | WTP | Australian Shelduck | 0.71 | 1.04 | 1.67 | 101.67 |
| 11 | WTP | Australian Shelduck | 0.67 | 0.96 | 1.74 | 101.00 |
| 12 | WTP | Australian Shelduck | 0.79 | 0.90 | 1.51 | 102.12 |
| 1 | VIC | Australian Shelduck | 1.28 | 1.19 | 1.14 | 100.66 |
| 2 | VIC | Australian Shelduck | 1.30 | 1.37 | 1.11 | 97.94 |
| 3 | VIC | Australian Shelduck | 1.26 | 1.33 | 1.13 | 98.80 |
| 4 | VIC | Australian Shelduck | 1.13 | 1.29 | 1.22 | 100.41 |
| 5 | VIC | Australian Shelduck | 1.06 | 1.29 | 1.25 | 100.77 |
| 6 | VIC | Australian Shelduck | 1.04 | 1.24 | 1.27 | 101.58 |
| 7 | VIC | Australian Shelduck | 1.05 | 1.14 | 1.25 | 102.63 |
| 8 | VIC | Australian Shelduck | 0.93 | 1.07 | 1.35 | 103.11 |
| 9 | VIC | Australian Shelduck | 0.81 | 1.00 | 1.51 | 102.78 |
| 10 | VIC | Australian Shelduck | 0.74 | 0.93 | 1.63 | 101.87 |
| 11 | VIC | Australian Shelduck | 0.72 | 0.95 | 1.66 | 101.88 |
| 12 | VIC | Australian Shelduck | 0.80 | 1.03 | 1.54 | 102.69 |
| 1 | SE | Australian Shelduck | 1.24 | 1.19 | 1.16 | 101.38 |
| 2 | SE | Australian Shelduck | 1.34 | 1.37 | 1.11 | 98.77 |
| 3 | SE | Australian Shelduck | 1.19 | 1.36 | 1.23 | 99.74 |
| 4 | SE | Australian Shelduck | 1.19 | 1.36 | 1.23 | 99.74 |
| 5 | SE | Australian Shelduck | 1.09 | 1.37 | 1.28 | 99.77 |
| 6 | SE | Australian Shelduck | 1.08 | 1.32 | 1.29 | 100.54 |
| 7 | SE | Australian Shelduck | 1.05 | 1.20 | 1.28 | 102.09 |
| 8 | SE | Australian Shelduck | 0.90 | 1.10 | 1.41 | 102.83 |
| 9 | SE | Australian Shelduck | 0.79 | 1.02 | 1.56 | 102.61 |
| 10 | SE | Australian Shelduck | 0.71 | 0.95 | 1.68 | 101.83 |
| 11 | SE | Australian Shelduck | 0.69 | 0.96 | 1.71 | 101.75 |
| 12 | SE | Australian Shelduck | 0.80 | 1.04 | 1.55 | 102.67 |
| 1 | MDB | Australian Shelduck | 1.39 | 1.05 | 1.04 | 101.28 |
| 2 | MDB | Australian Shelduck | 1.65 | 1.28 | 0.97 | 99.15 |
| 3 | MDB | Australian Shelduck | 1.80 | 1.42 | 0.97 | 97.82 |
| 4 | MDB | Australian Shelduck | 1.94* | 1.65* | 0.99 | **96.13** |
| 5 | MDB | Australian Shelduck | 1.81* | 1.74* | 1.05 | **95.18** |
| 6 | MDB | Australian Shelduck | 1.69 | 1.69 | 1.11 | 95.46 |
| 7 | MDB | Australian Shelduck | 1.48 | 1.44 | 1.11 | 99.35 |
| 8 | MDB | Australian Shelduck | 1.20 | 1.22 | 1.20 | 102.33 |
| 9 | MDB | Australian Shelduck | 0.97 | 1.05 | 1.33 | 103.20 |
| 10 | MDB | Australian Shelduck | 0.81 | 0.94 | 1.48 | 102.85 |
| 11 | MDB | Australian Shelduck | 0.76 | 0.92 | 1.54 | 102.61 |
| 12 | MDB | Australian Shelduck | 0.83 | 1.01 | 1.49 | 102.75 |
| 1 | WTP | Pacific Black Duck | 0.84 | 0.87 | 1.07 | 104.35 |
| 2 | WTP | Pacific Black Duck | 0.93 | 0.73 | 0.99 | 102.30 |
| 3 | WTP | Pacific Black Duck | 1.09 | 0.66* | 0.88 | 98.42 |
| 4 | WTP | Pacific Black Duck | 1.12 | 0.63* | 0.85 | **96.20** |
| 5 | WTP | Pacific Black Duck | 1.26 | 0.68* | 0.81 | **96.24** |
| 6 | WTP | Pacific Black Duck | 1.27 | 0.67* | 0.81 | **95.87** |
| 7 | WTP | Pacific Black Duck | 1.36 | 0.75 | 0.78 | 99.81 |
| 8 | WTP | Pacific Black Duck | 1.34 | 0.86 | 0.79 | 103.36 |
| 9 | WTP | Pacific Black Duck | 1.02 | 0.95 | 0.97 | 105.37 |
| 10 | WTP | Pacific Black Duck | 0.86 | 1.02 | 1.11 | 105.18 |
| 11 | WTP | Pacific Black Duck | 0.69 | 1.02 | 1.34 | 103.60 |
| 12 | WTP | Pacific Black Duck | 0.82 | 0.95 | 1.14 | 104.88 |
| 1 | VIC | Pacific Black Duck | 0.98 | 0.84 | 0.98 | 104.39 |
| 2 | VIC | Pacific Black Duck | 1.07 | 0.90 | 0.94 | 104.98 |
| 3 | VIC | Pacific Black Duck | 1.17 | 1.04 | 0.90 | 104.89 |
| 4 | VIC | Pacific Black Duck | 1.09 | 1.22 | 0.96 | 103.88 |
| 5 | VIC | Pacific Black Duck | 1.08 | 1.39 | 0.96 | 101.45 |
| 6 | VIC | Pacific Black Duck | 1.01 | 1.48* | 1.02 | **99.89** |
| 7 | VIC | Pacific Black Duck | 0.90 | 1.53* | 1.11 | **98.52** |
| 8 | VIC | Pacific Black Duck | 0.77 | 1.49* | 1.24 | **98.63** |
| 9 | VIC | Pacific Black Duck | 0.63 | 1.33 | 1.47 | 100.14 |
| 10 | VIC | Pacific Black Duck | 0.59 | 1.17 | 1.55 | 101.36 |
| 11 | VIC | Pacific Black Duck | 0.56 | 1.01 | 1.59 | 101.32 |
| 12 | VIC | Pacific Black Duck | 0.65 | 0.92 | 1.40 | 102.77 |
| 1 | SE | Pacific Black Duck | 1.00 | 0.73 | 0.92 | 102.02 |
| 2 | SE | Pacific Black Duck | 1.13 | 0.77 | 0.87 | 102.56 |
| 3 | SE | Pacific Black Duck | 1.26 | 0.89 | 0.83 | 103.70 |
| 4 | SE | Pacific Black Duck | 1.23 | 1.04 | 0.88 | 104.66 |
| 5 | SE | Pacific Black Duck | 1.21 | 1.24 | 0.91 | 103.49 |
| 6 | SE | Pacific Black Duck | 1.15 | 1.34 | 0.96 | 102.27 |
| 7 | SE | Pacific Black Duck | 1.01 | 1.42 | 1.06 | 101.27 |
| 8 | SE | Pacific Black Duck | 0.85 | 1.39 | 1.20 | 101.37 |
| 9 | SE | Pacific Black Duck | 0.70 | 1.22 | 1.37 | 102.67 |
| 10 | SE | Pacific Black Duck | 0.63 | 1.06 | 1.47 | 102.93 |
| 11 | SE | Pacific Black Duck | 0.58 | 0.91 | 1.52 | 101.92 |
| 12 | SE | Pacific Black Duck | 0.64 | 0.82 | 1.36 | 102.17 |
| 1 | MDB | Pacific Black Duck | 0.77 | 0.58** | 1.00 | **95.08** |
| 2 | MDB | Pacific Black Duck | 0.83 | 0.56** | 0.94 | **95.08** |
| 3 | MDB | Pacific Black Duck | 0.95 | 0.61* | 0.86 | 98.32 |
| 4 | MDB | Pacific Black Duck | 1.11 | 0.74 | 0.82 | 101.59 |
| 5 | MDB | Pacific Black Duck | 1.44 | 1.01 | 0.78 | 102.97 |
| 6 | MDB | Pacific Black Duck | 1.53 | 1.15 | 0.79 | 102.79 |
| 7 | MDB | Pacific Black Duck | 1.46 | 1.23 | 0.82 | 103.29 |
| 8 | MDB | Pacific Black Duck | 1.32 | 1.22 | 0.86 | 104.23 |
| 9 | MDB | Pacific Black Duck | 1.06 | 1.03 | 0.96 | 105.44 |
| 10 | MDB | Pacific Black Duck | 0.76 | 0.83 | 1.14 | 104.58 |
| 11 | MDB | Pacific Black Duck | 0.55 | 0.67 | 1.33 | 101.46 |
| 12 | MDB | Pacific Black Duck | 0.44* | 0.56* | 1.45 | 97.76 |
| 1 | WTP | Grey Teal | 0.90 | 0.93 | 0.73 | 100.19 |
| 2 | WTP | Grey Teal | 0.79 | 0.87 | 0.79 | 99.01 |
| 3 | WTP | Grey Teal | 0.65 | 0.80 | 0.86 | 95.85 |
| 4 | WTP | Grey Teal | 0.60* | 0.79 | 0.91 | 93.71 |
| 5 | WTP | Grey Teal | 0.50** | 0.73 | 1.04 | 89.39 |
| 6 | WTP | Grey Teal | 0.47*** | 0.70* | 1.09 | 86.41 |
| 7 | WTP | Grey Teal | 0.45*** | 0.74* | 1.18 | 83.97 |
| 8 | WTP | Grey Teal | 0.46*** | 0.76 | 1.21 | 84.84 |
| 9 | WTP | Grey Teal | 0.58* | 0.69* | 1.03 | 87.28 |
| 10 | WTP | Grey Teal | 0.68 | 0.63** | 0.90 | 84.91 |
| 11 | WTP | Grey Teal | 0.67 | 0.56*** | 0.89 | 80.14 |
| 12 | WTP | Grey Teal | 0.81 | 0.52*** | 0.73 | **76.85** |
| 1 | VIC | Grey Teal | 0.61* | 1.13 | 0.94 | 93.07 |
| 2 | VIC | Grey Teal | 0.56** | 1.03 | 1.01 | 91.36 |
| 3 | VIC | Grey Teal | 0.56** | 1.00 | 1.01 | 91.35 |
| 4 | VIC | Grey Teal | 0.53** | 0.97 | 1.03 | 89.58 |
| 5 | VIC | Grey Teal | 0.53** | 0.89 | 1.04 | 89.20 |
| 6 | VIC | Grey Teal | 0.53** | 0.83 | 1.04 | 88.55 |
| 7 | VIC | Grey Teal | 0.49** | 0.82 | 1.14 | 87.23 |
| 8 | VIC | Grey Teal | 0.47** | 0.79 | 1.21 | 86.40 |
| 9 | VIC | Grey Teal | 0.51** | 0.72* | 1.17 | 86.38 |
| 10 | VIC | Grey Teal | 0.57* | 0.66** | 1.05 | 86.00 |
| 11 | VIC | Grey Teal | 0.63 | 0.61** | 0.97 | **84.59** |
| 12 | VIC | Grey Teal | 0.74 | 0.58*** | 0.85 | **83.29** |
| 1 | SE | Grey Teal | 0.61* | 1.12 | 0.97 | 93.26 |
| 2 | SE | Grey Teal | 0.55** | 1.02 | 1.04 | 91.58 |
| 3 | SE | Grey Teal | 0.56** | 0.98 | 1.02 | 92.06 |
| 4 | SE | Grey Teal | 0.53** | 0.96 | 1.05 | 90.19 |
| 5 | SE | Grey Teal | 0.53** | 0.88 | 1.04 | 90.07 |
| 6 | SE | Grey Teal | 0.53** | 0.83 | 1.01 | 89.57 |
| 7 | SE | Grey Teal | 0.50** | 0.82 | 1.12 | 88.55 |
| 8 | SE | Grey Teal | 0.48** | 0.78 | 1.18 | 88.60 |
| 9 | SE | Grey Teal | 0.50** | 0.72* | 1.15 | 87.81 |
| 10 | SE | Grey Teal | 0.54* | 0.66** | 1.07 | 86.53 |
| 11 | SE | Grey Teal | 0.57* | 0.60*** | 0.99 | **84.38** |
| 12 | SE | Grey Teal | 0.65 | 0.56*** | 0.86 | **82.86** |
| 1 | MDB | Grey Teal | 0.65* | 1.22 | 1.00 | 93.55 |
| 2 | MDB | Grey Teal | 0.56* | 1.10 | 1.08 | 91.54 |
| 3 | MDB | Grey Teal | 0.56* | 1.02 | 1.04 | 92.22 |
| 4 | MDB | Grey Teal | 0.48** | 0.91 | 1.11 | 89.66 |
| 5 | MDB | Grey Teal | 0.51* | 0.87 | 1.04 | 91.88 |
| 6 | MDB | Grey Teal | 0.52** | 0.82 | 0.99 | 92.23 |
| 7 | MDB | Grey Teal | 0.48** | 0.81 | 1.08 | 91.14 |
| 8 | MDB | Grey Teal | 0.47** | 0.77 | 1.13 | 91.76 |
| 9 | MDB | Grey Teal | 0.40** | 0.67* | 1.21 | 89.15 |
| 10 | MDB | Grey Teal | 0.37*** | 0.60** | 1.25 | 86.25 |
| 11 | MDB | Grey Teal | 0.34*** | 0.51*** | 1.23 | **83.39** |
| 12 | MDB | Grey Teal | 0.37*** | 0.48*** | 1.10 | **84.71** |
| 1 | WTP | Pink-eared Duck | 0.92 | 0.86 | 0.69 | 99.07 |
| 2 | WTP | Pink-eared Duck | 0.77 | 0.92 | 0.79 | 98.34 |
| 3 | WTP | Pink-eared Duck | 0.68 | 0.93 | 0.84 | 96.46 |
| 4 | WTP | Pink-eared Duck | 0.63* | 0.96 | 0.89 | 94.64 |
| 5 | WTP | Pink-eared Duck | 0.55** | 0.91 | 0.99 | 91.92 |
| 6 | WTP | Pink-eared Duck | 0.51** | 0.88 | 1.04 | 89.49 |
| 7 | WTP | Pink-eared Duck | 0.48*** | 0.90 | 1.14 | 87.27 |
| 8 | WTP | Pink-eared Duck | 0.45*** | 0.85 | 1.23 | 85.22 |
| 9 | WTP | Pink-eared Duck | 0.48** | 0.77 | 1.19 | 84.26 |
| 10 | WTP | Pink-eared Duck | 0.57* | 0.69** | 1.02 | 84.29 |
| 11 | WTP | Pink-eared Duck | 0.60* | 0.60*** | 0.96 | **80.33** |
| 12 | WTP | Pink-eared Duck | 0.67 | 0.60*** | 0.85 | 84.77 |
| 1 | VIC | Pink-eared Duck | 0.72 | 1.24 | 0.85 | 95.09 |
| 2 | VIC | Pink-eared Duck | 0.61* | 1.21 | 0.95 | 91.60 |
| 3 | VIC | Pink-eared Duck | 0.53** | 1.14 | 1.02 | 88.40 |
| 4 | VIC | Pink-eared Duck | 0.51*** | 1.04 | 1.05 | 87.09 |
| 5 | VIC | Pink-eared Duck | 0.50*** | 0.92 | 1.06 | 85.97 |
| 6 | VIC | Pink-eared Duck | 0.51*** | 0.80 | 1.05 | 84.42 |
| 7 | VIC | Pink-eared Duck | 0.48*** | 0.74* | 1.13 | 81.51 |
| 8 | VIC | Pink-eared Duck | 0.45*** | 0.69** | 1.24 | 76.67 |
| 9 | VIC | Pink-eared Duck | 0.42*** | 0.66*** | 1.32 | **72.29** |
| 10 | VIC | Pink-eared Duck | 0.44*** | 0.67** | 1.29 | 75.81 |
| 11 | VIC | Pink-eared Duck | 0.49** | 0.64** | 1.17 | 78.93 |
| 12 | VIC | Pink-eared Duck | 0.56* | 0.69* | 1.06 | 86.94 |
| 1 | SE | Pink-eared Duck | 0.70 | 1.36 | 0.91 | 91.77 |
| 2 | SE | Pink-eared Duck | 0.60* | 1.30 | 1.01 | 88.34 |
| 3 | SE | Pink-eared Duck | 0.53** | 1.22 | 1.07 | 85.49 |
| 4 | SE | Pink-eared Duck | 0.50*** | 1.11 | 1.10 | 85.68 |
| 5 | SE | Pink-eared Duck | 0.49*** | 0.97 | 1.08 | 86.33 |
| 6 | SE | Pink-eared Duck | 0.49*** | 0.86 | 1.06 | 86.25 |
| 7 | SE | Pink-eared Duck | 0.46*** | 0.79 | 1.15 | 83.38 |
| 8 | SE | Pink-eared Duck | 0.40*** | 0.73* | 1.31 | 78.39 |
| 9 | SE | Pink-eared Duck | 0.38*** | 0.70** | 1.40 | **74.99** |
| 10 | SE | Pink-eared Duck | 0.38*** | 0.72* | 1.43 | 77.53 |
| 11 | SE | Pink-eared Duck | 0.41*** | 0.69** | 1.30 | 81.56 |
| 12 | SE | Pink-eared Duck | 0.49** | 0.74* | 1.13 | 89.48 |
| 1 | MDB | Pink-eared Duck | 0.73 | 1.57** | 0.98 | 86.73 |
| 2 | MDB | Pink-eared Duck | 0.69 | 1.50* | 1.01 | 85.31 |
| 3 | MDB | Pink-eared Duck | 0.64* | 1.38 | 1.04 | 84.74 |
| 4 | MDB | Pink-eared Duck | 0.60* | 1.25 | 1.06 | 86.76 |
| 5 | MDB | Pink-eared Duck | 0.57* | 1.11 | 1.02 | 89.48 |
| 6 | MDB | Pink-eared Duck | 0.54* | 0.97 | 1.01 | 90.85 |
| 7 | MDB | Pink-eared Duck | 0.47** | 0.86 | 1.11 | 88.91 |
| 8 | MDB | Pink-eared Duck | 0.38*** | 0.75 | 1.27 | 84.47 |
| 9 | MDB | Pink-eared Duck | 0.32*** | 0.69* | 1.44 | **79.13** |
| 10 | MDB | Pink-eared Duck | 0.32*** | 0.72 | 1.46 | **79.96** |
| 11 | MDB | Pink-eared Duck | 0.33*** | 0.74 | 1.42 | 83.98 |
| 12 | MDB | Pink-eared Duck | 0.46* | 0.89 | 1.18 | 91.62 |
